# 

**Published:** 1851-07

**Authors:** 


### CONTENTS

### OF NO. I.

---

### ORIGINAL COMMUNICATIONS.

#### ESSAYS AND CASES.

ART. I.—On the treatment of Dysentery. By JOHN HARDIN, M. D., of Louisville, Ky. A letter addressed to the editors of the West. Jour. Med. and Surg. - - - 1

ART. II.—Vital Statistics of Scott county, Ky., digested from the census as taken in 1850. By W. L. SUTTON, M. D., of Georgetown, Ky. - - - - - - - - - - 7

ART. III.—The Case of Sulphuretted Hydrogen Gas poisoning. By THEODORE S. BELL, M. D. - - - - - - 19

#### REVIEWS.

ART. IV.—Materia Medica and Therapeutics: with ample illustrations of Practice in all the departments of Medical Science, and very copious notices of Toxicology, suited to the wants of Medical Students and Practitioners. By THOMAS D. MITCHELL, A. M., M. D., Professor of the Theory and Practice of Medicine in the Philadelphia College of Medicine; formerly Professor of Chemistry and Pharmacy in the Medical College of Ohio; Professor of Chemistry and Materia Medica in the Medical Department of Transylvania University, Lexington, Ky.; Lecturer on Obstetrics and Diseases of Women and Children; Author of "Elements of Chemical Philosophy," "Hints to Students," &c., &c., &c. - - - - - - - - 37

SELECTIONS FROM FOREIGN AND HOME JOURNALS.

The American Medical Association, - - - - - - 61
Views of the Germans on the Operation of Turning, - - 84

ORIGINAL INTELLIGENCE.

American Medical Association, - - - - - - - 85
Smithsonian Institution, - - - - - - - - - 87
American Scientific Association, - - - - - - 88
Professor Bartlett, - - - - - - - - - - 89
Epidemics of Kentucky and Tennessee, - - - 89
Health of Louisville, - - - - - - - - - 90
Transactions of the College of Physicians of Philadelphia, - 90
Death of Dr. Samuel George Morton, - - - - - 90
Tracheotomy, - - - - - - - - - - - - 91
The Sulphuretted Hydrogen Gas case, - - - - - 91
Books Received, - - - - - - - - - - 91
Results of surgical operations in malignant diseases, - - 92

